# Internalizing/Externalizing Problems and Sensory Processing Alteration in Children Referred to Child Mental Health Centers

**DOI:** 10.3390/children12060664

**Published:** 2025-05-22

**Authors:** Macarena Valencia, Ana D’Ocon, Raquel Plata, Sandra Simó, María José Cantero

**Affiliations:** 1 Bio-Gipuzkoa Institute of Health Research, 20014 Gipuzkoa, Spain; 2 Department of Basic Psychology, University of Valencia, 46010 Valencia, Spain; 3 Department of Developmental and Educational Psychology, University of Valencia, 46010 Valencia, Spain

**Keywords:** internalizing problems, externalizing problems, sensory processing alteration, sensory processing disorder (SPD), middle childhood, childhood problems, child mental health center

## Abstract

Background/Objectives: Internalizing and externalizing problems have been associated with sensory processing (SP) alteration, where severe alterations are equivalent to sensory processing disorder (SPD). This study aims to examine the relationship between childhood problems and the degree of SP alteration in children referred to a child mental health center (CMHC). Methods: The study included sixty-four children (44 boys and 20 girls), aged 6 to 8 years, referred to a CMHC in Gipuzkoa, Spain. Parents completed the Child Behavior Checklist (CBCL/6–18) and the Child Sensory Profile (CSP-2). Analyses of variance and qualitative analyses were conducted to compare T-scores of childhood problems across SP alteration groups (no alteration, mild, moderate, and severe). Results: Of the participants, 31.3% (n = 20) exhibited a severe SP alteration and obtained significantly higher scores than those without SP alteration on the Withdrawn/Depressed and Attention, Social, and Thought Problems syndrome scales, as well as on the Internalizing Problems and Total Problems scales. Furthermore, children with severe SP alteration scored within the clinical range on the Anxious/Depressed and Attention Problems scales. Mean scores for Internalizing, Externalizing and Total Problems reached the clinical threshold when the SP alteration was classified as moderate or severe. These findings indicate that SP alteration is associated with increased internalizing and externalizing problems in children referred to the CMHC, with clinical levels observed particularly in cases of moderate to severe SP alteration. Conclusions: This study highlights the importance of including SP assessment in the diagnostic evaluation of childhood mental health problems.

## 1. Introduction

In recent decades, research in developmental psychopathology has focused on understanding the pathways through which internalizing and externalizing problems in childhood may lead to difficulties in social adjustment and mental health during adolescence and adulthood [1,2]. The present study aims to contribute to a deeper understanding of these problems in childhood by incorporating sensory processing (SP) as developmental variable relevant to the school-age period. Internalizing problems include symptoms such as anxiety, depression, social withdrawal, somatic complaints, phobias, and feelings of guilt, whereas externalizing problems are characterized by aggressive behavior or non-compliance with rules [3]. According to Forns et al. [4] attention-deficit/hyperactivity disorder (ADHD) is also considered part of the externalizing domain.

In a meta-analysis on the prevalence and comorbidity of mental disorders in children aged 1–7 years, Vasileva et al. [5] reported that externalizing problems—including oppositional defiant disorder, ADHD, and conduct disorder—had a pooled prevalence of 10%. For internalizing problems, such as depression and anxiety disorders, the pooled prevalence was 9.6%. Moreover, these disorders may have a higher likelihood of co-occurrence, and the interaction between internalizing and externalizing problems evolves throughout childhood [6].

Both internalizing and externalizing problems have been associated with a wide range of adverse outcomes during childhood and adolescence, including difficulties in cognitive functioning, poor academic performance [7], and deficits in self-regulation [2,6]. These difficulties may increase the risk of developing major depression or engaging in suicidal behaviors during adolescence and adulthood [8]. Therefore, early identification of these problems and analysis of the factors associated with their developmental trajectories should be considered a public health priority. In this regard, several studies have examined the relationship between internalizing and externalizing problems and SP [9,10,11].

SP is defined by Dunn [12] as the ability to organize and integrate sensory information from both the external environment and from one’s own body to produce an adaptive response. To further understand how children process sensory information and how it influences their behavior, the “Model of Sensory Processing” [13] proposes an interaction between neurological thresholds and behavioral self-regulation. According to this model, individuals respond differently to sensory information based on their response speed (low vs. high threshold) and how they regulate sensory stimuli (low vs. high self-regulation). The intersection of these two continua results in four basic SP patterns that, according to Dunn [13], influence behavior organization and emotion regulation: sensation seeking (high thresholds and active self-regulation strategy), sensation avoiding (low thresholds and active self-regulation strategy), sensory sensitivity (low thresholds and passive self-regulation strategy), and low registration (high thresholds and passive self-regulation strategy). Sensation-seeking children have been shown to have elevated sensory thresholds and actively seek stimulation. They have been observed to derive pleasure from intense sensory experiences and tend to exhibit high levels of activity. Sensation-avoiding children have low thresholds and actively limit sensory input. They often withdraw from stimulation and prefer routines to maintain control. Sensory-sensitive children have been shown to have low thresholds but respond passively to sensory input. They are easily overwhelmed and do not take steps to avoid it, which can lead to anxious or distracted behavior. Finally, low-registration children exhibit high thresholds and passive responses. These children may not notice sensory cues and often appear disengaged or slow to respond, requiring external prompts to stay involved [12].

When SP is disrupted, it leads to what is known as sensory processing disorder (SPD), characterized by dysfunctional processing and/or organization responses to sensory information, which impairs daily routines and functional activities [14]. An estimated 12–20% of children in the United States exhibit atypical SP and require specific strategies to adapt their behavior to everyday life [15]. A study conducted in Spain by Galiana et al. [16] developed a classification system to assess sensory profile alterations in children, based on Dunn’s model. This system categorizes children into four levels of SP alteration: no alteration, mild, moderate, and severe, based on the intensity and impact of SP difficulties on daily functioning. This classification allows for a clearer identification of sensory difficulties, facilitating the development of appropriate intervention strategies. The aim of their study was to assess the prevalence and severity of SPD in Spanish children aged 5–9 years, and they found that 15.9% met the criteria for severe SP alteration (SPD), while 10.5% presented a moderate alteration (SPD risk).

SPD is a condition that may occur independently of other childhood disorders [10]. However, it is often associated with neurodevelopmental disorders [17], particularly in autism spectrum disorder (ASD) [18]. Furthermore, SPD can impact mental health and be an early indicator of psychopathology in childhood [10,19,20]. Despite this body of evidence, SPD is not recognized in diagnostic classification systems such as the DSM-5-TR or ICD-11. SP alteration is only included as one of the criteria for restrictive and repetitive patterns of behavior, interests, or activities in the diagnosis of ASD (DSM-5-TR) [21].

Difficulties in SP can affect one or more sensory systems, which in turn may manifest in various behaviors. This hinders the accurate understanding and diagnosis of a given case, making it challenging to design an appropriate treatment strategy tailored to the child’s needs [18]. At school age, children with SPD may experience difficulties with attention [22], social interactions with peers, and the development of motor skills or motor planning [23]. In addition, these difficulties can lead to feelings of inadequacy, social isolation, and emotional outbursts [12,23].

More specifically, regarding the relationship between internalizing problems and SPD [24], an association between sensory over-responsivity (SOR; low threshold, sensory hyperreactivity) and anxiety in children aged 6 to 10 years old with diagnoses of ASD and ADHD and typically developing children (TYP) was identified. On the other hand, Boterberg and Warreyn [25] found that heightened SP sensitivity (low threshold and passive self-regulation strategies) was linked to increased internalizing problems in children without neurodevelopmental disorders. Similarly, Dean et al. [26] demonstrated that sensation-seeking behavior was negatively associated with depressive symptoms and positively correlated with resilient behaviors. Regarding externalizing behavior, studies have shown that heightened sensory reactivity (low threshold) correlates with increased impulsivity [27] and that sensory avoidance behaviors are related to increased externalizing behaviors, such as hyperactivity, aggressiveness, and conduct problems [26]. These findings suggest that psychological problems in childhood may, in fact, mask underlying SP difficulties [9].

Studying the relationship between SPD in middle childhood and childhood problems holds significant clinical relevance, as it provides a deeper understanding of the processes underlying psychological maladjustment in children. This knowledge facilitates preventive interventions and helps reframe therapeutic strategies to address specific sensory issues. The present study aims to analyze the relationship between internalizing and externalizing problems in childhood and the degree of SP alteration in children aged 6 to 8 years who were referred to a child mental health center (CMHC). The central research question guiding this study is: Do children referred to a CMHC for psychological and/or psychiatric assessment with severe or moderate SP alterations (SPD or at risk of SPD) exhibit higher levels of childhood problems compared to children with no SP alterations or only mild alterations?

The participants’ sex was controlled for, given the established differences between boys and girls in this context. Specifically, research suggests that girls are more likely to exhibit internalizing problems, while boys tend to display more externalizing problems, particularly during adolescence [6]. Regarding SPD, however, evidence is contradictory: while males are more likely to develop SPD than females [10], gender has not been identified as a significant predictor of SPD [9,28].

## 2. Materials and Methods

### 2.1. Participants

A total of 64 children aged 6 to 8 years (M = 7.19, SD = 0.71) participated in the study. They were referred by pediatricians to the CMHC of the Mental Health Network of Gipuzkoa, in the Basque Country (Spain). The sample consisted of 68.8% boys (n = 44) and 31.3% girls (n = 20). Regarding school grade, 15.6% (n = 10) were in first grade, 56.3% (n = 36) in second grade, and 28.1% (n = 18) in third grade. In terms of family income, 11% (n = 7) reported annual income below EUR 12,000; 26.6% (n = 17) between EUR 12,000 and EUR 23,000; 23.4% (n = 15) between EUR 24,000 and EUR 35,000; 18.8% (n = 12) between EUR 36,000 and EUR 50,000; and 17.2% (n = 11) reported income above EUR 50,000.

Participants met the following inclusion criteria: (1) referred to a CMHC and undergoing psychological and/or psychiatric diagnostic assessment, (2) no diagnosis of moderate-to-severe language disorders, autism spectrum disorder, or psychosis, (3) no more than four consultations with mental health professionals at the CMHC, (4) adequate proficiency in the Spanish language.

### 2.2. Instruments

Child Behavior Checklist—Parent Version (CBCL/6–18) [3]. The CBCL comprises 113 items with three response options (0 = not true, 1 = somewhat or sometimes true, 2 = very true or often true) that assess how frequently parents have observed specific behaviors in their children aged 6 to 18 years during the past six months. The syndrome scales are divided into eight clinical scales (Anxious/Depressed, Withdrawn/Depressed, Somatic Complaints, Social Problems, Thought Problems, Attention Problems, Rule-Breaking Behavior, and Aggressive Behavior), two composite scales (Externalizing Problems and Internalizing Problems) and one Total Problems scale.

For each scale, participants were classified into three categories based on their T-scores: non-clinical, clinical borderline range, and clinical range. Clinical cutoffs followed the Achenbach System of Empirically Based Assessment (ASEBA) guidelines [29]. The ASEBA is a comprehensive system for assessing behavioral and emotional functioning across age groups, using tools like the Child Behavior Checklist (CBCL) for clinical and research purposes. Higher T-scores indicate greater severity of symptoms. For syndrome scales, T-scores ≥ 70 indicated the clinical range, scores from 65 to 69 the clinical borderline range, and scores ≤ 64 were considered within the non-clinical range. For the composite scales and Total Problems scale cutoff points were more conservative: T-scores ≥ 64 were categorized as clinical, 60–63 as clinical borderline, and ≤59 as in the non-clinical range. These thresholds are based on normative samples and allow for standardized interpretation of behavioral and emotional difficulties [29].

The reliability of the Spanish version ranged from α = 0.71 to α = 0.87 [30]. In the present sample, Cronbach’s alpha values were as follows: 0.82 for the Anxiety/Depression scale, 0.77 for Withdrawal/Depressed, 0.77 for Somatic Complaints, 0.53 for Social Problems, 0.74 for Thought Problems, 0.71 for Attention Problems, 0.60 for Rule-Breaking Behavior, and 0.87 for Aggressive Behavior. For the composite scales, alpha values were 0.89 for Internalizing Problems, 0.90 for Externalizing Problems, and 0.92 for the Total Problems scale.

Spanish adaptation of Child Sensory Profile-2 (CSP-2) [28,31]. An adapted version of Dunn’s Sensory Profile-2 parent questionnaire was used to assess SP skills and their impact on functional performance in daily life in children aged 3 to 14 years. The instrument includes 86 items assessing six SP areas (visual, auditory, tactile, movement, oral, and body position), three behavioral sections (conduct, social–emotional responses, and attention responses), and four basic SP patterns (low registration, sensation seeking, sensory sensitivity, and sensation avoiding). Items are rated on a 5-point Likert scale (1 = never/almost never to 5 = always/almost always), based on how frequently parents observe each behavior. Each SP pattern is classified according to the following scoring system: Typical Performance (TP, ±0 SD), Probable Difference (PD, ±1 SD), and Definitive Difference (DD, ±2 SD) [28]. A greater number of PD or DD classifications indicates a more severe SP alteration. Following this model, Galiana et al. [16] developed a four-group classification system based on the number of patterns that fall into each category (TP, PD, or DD): (1) no SP alteration, (2) mild SP alteration, (3) moderate SP alteration, and (4) severe SP alteration. For example, if all four patterns are classified as TP, the case is classified into group 1. In contrast, if one pattern is TP, two are PD, and one is DD, the case is classified into group 4. See Galiana et al. [16] for a detailed description of this classification system.

For each of the measures provided by the SP-2, reliability coefficients in the Spanish normative sample ranged from adequate to excellent (α = 0.74 to 0.87), while inter-informant agreement ranged from 0.73 to 0.89 [28]. High internal consistency was also observed in our sample for the four basic SP patterns, with Cronbach’s alpha values of 0.86 for the Sensation-Seeking Pattern, 0.87 for Sensation Avoiding, 0.87 for Sensory Sensitivity, and 0.89 for Low Registration.

### 2.3. Procedure

This study was approved by the Clinical Research Ethics Committee of the Gipuzkoa Health Area at the University Hospital of Donostia-San Sebastián (GAR-APE-2019-01). The research was presented to professionals within the Mental Health Network, and the inclusion criteria for participants were clearly specified. A non-probabilistic convenience sampling method was used. Selected children and their families were informed about the objectives of the study and provided written informed consent. The data collection was carried out at the CMHCs by mental health professionals.

### 2.4. Data Analysis

All analyses were performed using SPSS Statistics, version 26.0. Contingency tables (χ^2^) and *t*-tests were used to control for sex differences. To examine the relationship between childhood problems and the degree of SP alteration, two types of analyses were performed: (1) univariate ANOVAs, with the four levels of SP alteration (no alteration, mild alteration, moderate alteration, and severe alteration) as the independent variable and scores on the different CBCL problems scales as the dependent variables. A Bonferroni correction was applied to control for Type I error due to multiple comparisons and post hoc Bonferroni tests were conducted to assess pairwise group differences. (2) Qualitative analyses of T-scores based on the clinical cutoffs (non-clinical, clinical borderline, and clinical) were also conducted [29].

Given that participants were recruited from CMHCs, achieving the statistically recommended sample size was not feasible due to clinical and logistical constraints typical of this population. For a one-way ANOVA with four groups, a total sample size of N = 64, an alpha level of 0.05, and a desired power of 0.80, the G*Power analysis indicated that the study is powered to detect effect sizes of f ≥ 0.42 (d ≈ 0.84). According to Cohen’s conventions, this represents a large effect size, suggesting that the current sample is sufficient to detect large effects.

## 3. Results

In the present study, 42.19% (n = 27) of participants showed no alteration in SP, 17.19% (n = 11) showed mild alteration, 9.38 (n = 6) showed moderate alteration, and 31.25% (n = 20) showed severe alteration. To control for the variable sex, contingency tables were made for the variable SP alteration, and independent samples *t*-tests were conducted for the variables related to childhood problems. No statistically significant differences were observed in the severity of SP alteration by sex (χ^2^ (3, N = 64) = 0.89 *p* = 0.83). Likewise, no statistically significant differences were observed between boys and girls in relation to childhood problems (see Table 1). The Bonferroni correction was not applied, as none of the comparisons reached statistical significance.

However, when qualitative analyses were performed based on T-scores for CBCL Syndrome scales, Attention Problems reached clinical borderline values in the total sample (T = 66.49), as well as in the subsamples of boys (T = 65.67) and girls (T = 68.25). Additionally, the mean T-score for Anxious/Depressed symptoms reached clinical borderline levels exclusively in girls (T = 67.50). No other syndrome scales reached clinical borderline values in the total sample or in either sex subgroup.

Notably, scores for Internalizing Problems reached the clinical borderline range (T-score between 60 and 63) in the total sample (T = 63.90) and in the subsample of boys (T = 62.72). In girls, the average score fell within the clinical range (T = 66.45). Regarding Externalizing Problems, mean scores were within the clinical borderline range for the total sample (T = 60.63), as well as for boys (T = 60.40) and girls (T = 61.15). In terms of total childhood problems, mean scores reached the clinical borderline range across all cases.

Table 2 shows the results of the ANOVAs examining the relationship between CBCL syndrome scales and the level of SP alteration. The table includes mean scores and SD for each group based on the level of SP alteration, post hoc Bonferroni comparisons, and effect sizes (Cohen’s d). Both the ANOVAs and the post hoc comparisons applied the Bonferroni correction to control for Type I error.

**Figure 1:**
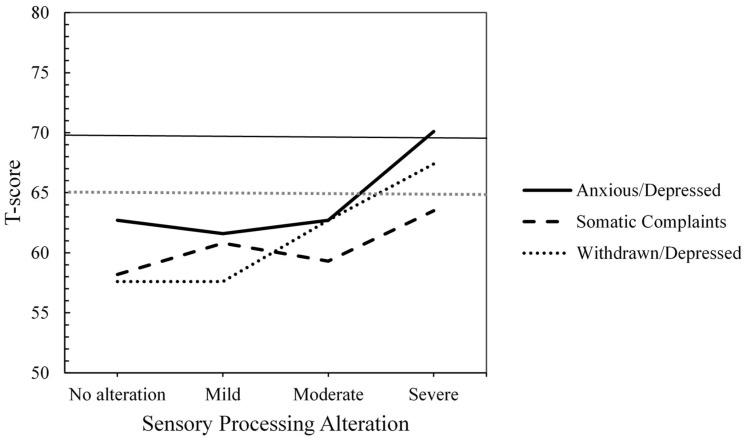
Mean T-scores by Group of Sensory Processing Alteration for Internalizing Problems (T-Score Ranges: ≥70 = Clinical, 65–69 = Clinical Borderline, ≤64 = Non-Clinical).

Regarding Internalizing Problems, no statistically significant differences were observed in Anxious/Depressed problems (F(3, 59) = 2.66, *p* = 0.057, η^2^ₚ = 0.119) based on the severity levels of SP alteration. However, the effect size was moderate to large, suggesting potentially meaningful differences that may not have reached significance due to sample size or variability. On the other hand, qualitative analyses based on T-scores revealed that children with severe SP alteration had mean Anxiety/Depression scores within the clinical range (T-score ≥ 70; see Figure 1). In contrast, children with no SP alteration, as well as those with mild and moderate alterations, showed scores within the non-clinical range (T-score < 65).

Statistically significant differences were observed in Withdrawn/Depressed problems (F(3, 59) = 5.46, *p* = 0.002 (Bonferroni correction: α = 0.006), η^2^ₚ = 0.217) based on the severity of SP alteration with a large effect size. Children with no SP alteration (mean difference = −9.78, SE = 2.59, *p* = 0.002 (Bonferroni correction: α = 0.008), d = −1.15, 95% CI [−16.85, −2.70]) scored significantly lower on Withdrawn/Depressed problems than children with severe SP alteration. When analyzing the clinical levels defined by the CBCL, children with severe SP alteration showed clinical borderline scores (T-score between 65 and 69) on this scale (see Figure 1), while the other groups fell within the non-clinical range (T-score < 65).

Finally, no significant differences were found in Somatic Complaints based on the severity of the SP alteration (F(3, 59) = 1.73, *p* = 0.170, η^2^ₚ = 0.081). Nevertheless, the effect size was moderate, suggesting that the severity of SP alteration may still have a meaningful, though statistically non-significant, association with somatic symptoms. At the clinical level, all groups had T-scores within the non-clinical range (T-score < 65; see Figure 1).

**Figure 2:**
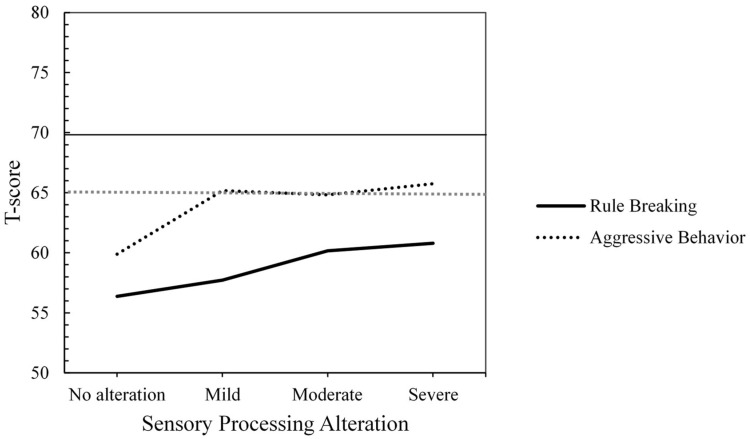
Mean T-scores by Group of Sensory Processing Alteration for Externalizing Problems (T-Score Ranges: ≥ 70 = Clinical, 65–69 = Clinical Borderline, ≤64 = Non-Clinical).

Regarding Externalizing Problems, no statistically significant differences were found in Rule-Breaking Behavior (F(3, 59) = 1.71, *p* = 0.175, η^2^ₚ = 0.080) or in Aggressive Behavior (F(3, 59) = 2.04, *p* = 0.118, η^2^ₚ = 0.094) based on the severity levels of SP alteration. In both cases, the effect size was moderate. T-Score analyses for Rule-Breaking Behavior indicated a stable non-clinical profile. However, for Aggressive Behavior, the average T-scores increased to the clinical borderline range (T-score between 65 and 69) starting from a mild alteration in SP (see Figure 2).

**Figure 3:**
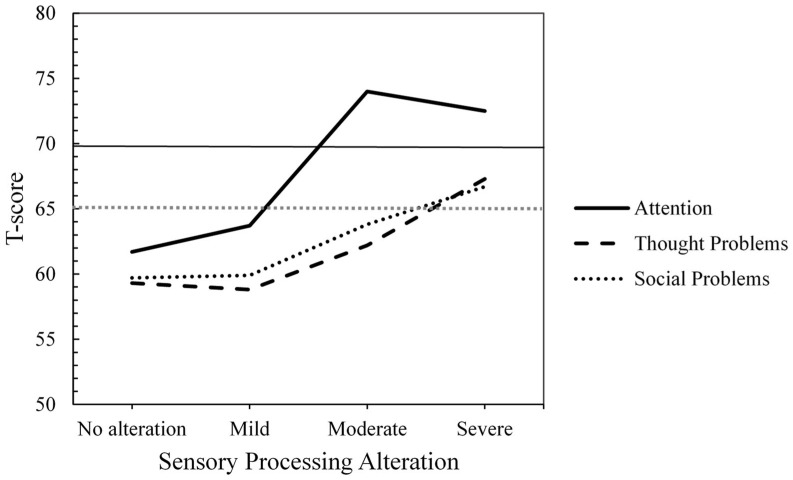
Mean T-scores by Group of Sensory Processing Alteration for Attention, Thought, and Social Problems (T-Score Ranges: ≥70 = Clinical, 65–69 = Clinical Borderline, ≤ 64 = Non-Clinical).

With respect to the other child problems evaluated, after applying the Bonferroni correction, no statistically significant differences were found in Social Problems (F(3, 59) = 4.10, *p* = 0.01 (Bonferroni correction: α = 0.006), η^2^ₚ = 0.173) or Thought Problems (F(3, 59) = 4.23, *p* = 0.009 (Bonferroni correction: α = 0.006), η^2^ₚ = 0.177). However, the effect sizes were large in both (η^2^ₚ > 0.14), suggesting that the severity of SP alteration may have a meaningful impact on these domains, even though the results did not reach the adjusted threshold for statistical significance. Qualitative T-score analyses indicated that only the severe SP alteration group reached the clinical borderline range in both Social Problems and Thought Problems (see Figure 3).

In contrast, statistically significant differences were observed in Attention Problems based on the severity of the SP alteration (F(3, 59) = 5.68, *p* = 0.002 (Bonferroni correction: α = 0.006), η^2^ₚ = 0.224). Children with no SP alteration scored significantly lower than those with severe alteration (mean difference = −10.82, SE = 3.02, *p* < 0.01 (Bonferroni correction: α = 0.008), d = −1.05, 95% CI [−19.07, −2.58]) with a large effect size. No significant differences were found between the non-altered and moderately altered SP groups in Attention Problems. However, the effect size was large (mean difference = −12.30, SE = 4.55, *p* = 0.054, d = −1.11, 95% CI [−24.72, +0.13]). Qualitative T-score analyses of attention problems showed that children with moderate and severe SP alteration reached the clinical range (T-score ≥ 70; see Figure 3).

Table 3 shows the results of the ANOVAs examining the relationship between CBCL composite scales (Internalized and Externalized Problems) and Total Problems scale by the level of SP alteration. Both the ANOVAs and the post hoc comparisons were adjusted using the Bonferroni correction to control for Type I error. For Internalizing Problems (F(3, 59) = 5.14, *p* = 0.003 (Bonferroni correction: α = 0.017), η^2^ₚ = 0.207) and Total Problems (F(3, 59) = 3.79; *p* = 0.015 (Bonferroni correction: α = 0.017), η^2^ₚ = 0.162), significant differences were observed between SP groups. Children with severely altered SP scored significantly higher on Internalizing Problems (mean difference = −9.44, SE = 2.49, *p* = 0.002 (Bonferroni correction: α = 0.008), d = −1.14, 95% CI [−16.24, −2.65]) than children with no SP alteration (see Table 3). However, for Total Problems, pairwise comparisons did not reach statistical significance (mean difference = −9.12, SE = 3.28, *p* = 0.044 (Bonferroni correction: α = 0.008), d = −1.23, 95% CI [−18.08, −0.15]) although the effect size was large (d > 0.50). Regarding Externalizing Problems, no differences were found between groups (F(3, 59) = 2.90; *p* = 0.042 (Bonferroni correction: α = 0.017), η^2^ₚ = 0.129) although a moderate-to-large effect size was observed. These findings suggest that the difference between groups may hold clinical or practical significance, despite not meeting the conventional threshold of η^2^ₚ = 0.14 typically associated with a large effect size.

**Figure 4:**
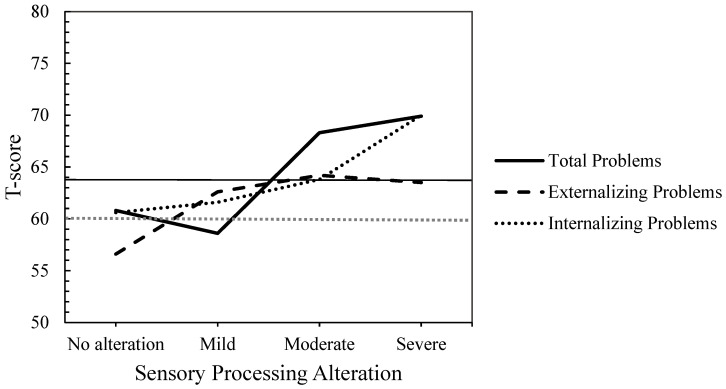
Mean T-scores by Group of Sensory Processing Alteration for Total, Externalizing, and Internalizing Problems (T-Score Ranges: ≥ 64 = Clinical, 60–63 = Clinical Borderline, ≤59 = Non-Clinical).

On the other hand, qualitative analyses based on T-scores from the composite and total scales revealed that scores in the clinical range were observed in participants with moderate and severe SP alteration for Externalizing and Total Problems and in those with severe SP alterations for Internalizing Problems (see Figure 4). Furthermore, scores reached the clinical borderline range even in cases of mild alteration (for Internalizing and Externalizing Problems) or no alteration at all (for Internalizing and Total Problems).

## 4. Discussion

This study aims to contribute to the field of child mental health by analyzing the role of SP alterations in childhood problems. Specifically, it investigates the association between SP difficulties and the presence of childhood problems in children aged 6 to 8 years, who have been referred to a mental health center due to the manifestation of externalizing and/or internalizing symptoms.

On the one hand, regarding SP, 31.3% of the participants in this study exhibited severe SP alteration, which may be considered consistent with a diagnosis of SPD [16]. In contrast, 9.38%, 17.9%, and 42.19% of participants presented moderate (indicative of SPD risk), mild, and no alteration, respectively. These findings contribute empirical data on the prevalence of SP in a treatment-referred pediatric population and enhance our understanding of the relationship between SP and childhood problems. A study by Galiana et al. [16], conducted in six public primary schools in Spain with children aged 5 to 9 years who had not been referred to a CMHC, revealed lower rates of SP alteration: 15.9% (severe), 10.5% (moderate), 11.1% (mild), and 62.5% (no alteration). When comparing our results with those reported by Galiana et al., it can be observed that the prevalence of severe SP is approximately twice as high in children with symptoms of childhood problems compared to their non-referred peers (31.3% vs. 15.9%). Notably, no sex-based differences were observed in our sample, which contrasts with previous studies reporting higher SPD prevalence in boys from non-clinical populations [10].

On the other hand, regarding childhood problems, T-scores within the clinical range were observed on the Internalized Problems and Total Problems scales. Although the differences between boys and girls did not reach statistical significance, an examination of the T-scores reveals that girls scored within the clinical range in the Internalizing dimension, with Anxiety/Depression problems accounting for the sex-based difference. This result accords with previous studies [6].

A key contribution of this study is the statistical and qualitative analysis of the association between childhood problems and SP alteration. From a statistical standpoint, a severely altered SP is associated with Withdrawn/Depressed and Attention Problems. However, in children referred to a CMHC, severe SP alteration is not associated with specific Externalizing Problems such as Aggressive or Rule-Breaking Behavior.

The qualitative analysis of children’s problem scores based on CBCL clinical parameters provides a complementary perspective that enriches the quantitative analysis of differences among SP-altered groups. Children whose scores fall within the clinical or clinical borderline ranges on most CBCL dimensions show moderate or severe SP alteration. This level of clinical dysfunction underscores the comorbidity between internalizing/externalizing problems and altered SP [24,26,32]. Within the Internalizing Problems dimension, the Anxious/Depressed problems and Withdrawn/Depressed problems scores fall within the clinical or clinical borderline range, respectively, only when the SP is severely altered. Specifically, during school age, the severity of SP difficulties plays an essential role in the clinical intensification of depressive and anxious symptoms. In this regard, the literature has consistently described that children with extreme SP patterns (hypo- or hyperresponsivity) often experience difficulties in modulating emotional and behavioral responses [20], which are in turn related to depressive symptoms [33], as well as high levels of anxiety, negative affect, and shyness [25]. Furthermore, SP difficulties have also been linked to clinically significant levels of anxiety in adulthood [34]. In relation to the Externalizing Problems dimension, only the scores on Aggressive Behavior problems fall within a clinical borderline range when the SP is severely altered. However, the overall Externalizing Problems score reaches a clinical range when the SP is moderately impaired. These results could be further clarified by examining the relationship between the SP functioning (visual, auditory, tactile, etc.) and specific externalizing problems [32].

It is noteworthy that as the SP reaches a moderate level of alteration (risk of SPD), attention problems move into the clinical range. That is, as sensory alteration increases, the clinical severity of attention problems scales up. These findings support those reported in previous studies of children with ADHD, demonstrating a significant relationship between this disorder and SP problems [22,33]. Furthermore, other studies have also linked attention and hyperactivity problems with sensory seeking [35], avoidant and sensitive patterns [36], and attention deficit with low-registration pattern [37]. In this regard, SP problems could be considered as a risk biomarker for the subsequent development of ADHD [17].

Different studies report that children with SPD have greater difficulties in performing daily activities, social participation, cognition, academic performance [38], temperament regulation [39], and sleep [40]. In this respect, our findings support the notion that SPD (severe SP alteration) is associated with clinical borderline scores on social problems (e.g., poor coordination and clumsiness, accident proneness, difficulties in relationships with peers, jealousy, feelings of loneliness, etc.) and thought problems (e.g., bizarre thoughts, sleep problems, tics or nervous movements, behaviors such as nose or skin picking, self-injury, etc.).

It bears noting that the school-age period represents a critical developmental stage characterized by significant neurodevelopmental changes. These changes may contribute to the emergence of psychiatric symptoms in children, particularly those exposed to adverse environments, thereby complicating their social adaptation [41]. Consequently, conducting a comprehensive assessment of the sensory profile in school-age children is essential to support their adaptation and prevent clinical distress. Diagnostic manuals such as DSM-5TR or ICD-10 do not include SP as a relevant construct to be taken into account in child psychiatric diagnoses, with the exception of ASD, in which it is recognized as a diagnostic criterion (criterion B.4) [21]. An exception is the DC: 0–5 manual [42], which explicitly recognizes SPD as a diagnosable condition in children aged 0–5 years. This reveals a gap that limits the formal recognition and diagnosis of SPD in school-age children, despite its evident clinical relevance. The present findings underscore the need for child mental health professionals to address SP difficulties as part of comprehensive diagnostic and intervention strategies.

Our findings are consistent with those of Gouze et al. [10], who emphasized that externalizing and internalizing problems in childhood may stem from underlying SP difficulties that often go undetected. In light of this, it might be time to consider SP as a potential biomarker, as proposed by Harrison et al. [19], to aid in differentiating between specific types of mental disorders. SP profiles could serve as a valuable tool for distinguishing both between and within mental disorders diagnoses, contributing to the identification of more homogeneous disorder subgroups [19] and enabling the development of more individualized and effective therapeutic interventions.

A limitation of this study is the relatively small sample size, which constrains statistical power to detect small-to-moderate effect sizes. While large effects were identified, non-significant results should be interpreted with caution, as they may reflect insufficient power rather than a true lack of effect. The reduced sample size is partially attributable to the clinical nature of the sample, as participants were recruited from CMCHs, where practical and ethical constraints often limit the availability of eligible individuals. Despite this, the study found strong associations between SP alterations and internalized problems and attention difficulties. The large effect sizes suggest that these relationships are not only statistically significant but also clinically relevant. These results align with and reinforce prior research in the field, highlighting the relevance of SP alteration in the context of child psychopathology. To enhance statistical power and generalizability, future studies could include a comparison group of children not referred to CMHCs, which would allow for more precise estimation of group differences and strengthen the interpretation of findings.

In conclusion, this study underscores the relevance of including SP assessment in the diagnostic evaluation of childhood mental health problems, primarily due to the following reasons:

(1) The similarity in the clinical manifestation of SP symptoms and other mental disorders at school age. For instance, some symptoms of ADHD, such as inattention, distractibility, and/or hyperactivity, are also commonly observed in SP problems. The interpretation of children’s behaviors for diagnostic purposes is crucial, as diagnostic labels inform decision making regarding service delivery, the choice of clinical interventions, and the application of evidence-based practices [32]. This understanding could provide families with a possible neurological explanation for their child’s disruptive behaviors [9].

(2) Their impact on children’s and their families’ lives is significant. The child’s environment (family and school) is a determining factor in both diagnosis and intervention in cases of SPD [43]. Regarding diagnosis, as indicated by the DC- 0–5, a necessary criterion of SPD is that the disorder’s symptoms affect the functioning of both the child and their family. From an intervention perspective, it is essential not only to support the child in managing of SP but also to address the environment and design activities that promote their daily participation [39]. In this regard, future studies would benefit from incorporating parental variables to better understand the mediating role of parents in the relationship between SP difficulties and childhood problems.

Addressing children’s mental health problems without considering and understanding potential sensory difficulties can undermine the effectiveness of a given treatment. Understanding the sensory and behavioral profile of children can be highly beneficial for professionals working with them in various contexts (health, school, family), as it can help organize and modulate the adult’s relationship with the child, provide appropriate strategies or routines to meet sensory needs, and offer more opportunities for successful engagement [12].

## Figures and Tables

**Table 1:** Means and Standard Deviations of T-scores for CBCL Syndrome, Composite, and Total Scales, with t-Values for Sex Comparisons.

		Sex		
CBCL	Total (n = 63) M (SD)	Male (n = 43) M (SD)	Female (n = 20) M (SD)	t (p)
Syndrome Scale				
Anxious/Depressed	64.73 (10.27)	63.44 (10.10)	*67.50* (10.41)	−1.47 (0.15)
Withdrawn/Depressed	61.03 (9.54)	60.42 (9.73)	62.35 (9.20)	−0.75 (0.46)
Somatic Complaints	60.35 (8.12)	60.02 (8.44)	61.05 (7.55)	−0.46 (0.64)
Rule-Breaking Behavior	58.30 (7.00)	57.95 (7.50)	59.05 (5.86)	−0.58 (0.57)
Aggressive Behavior	63.05 (9.04)	63.42 (9.54)	62.25 (8.01)	0.48 (0.64)
Attention Problems	*66.49* (11.17)	*65.67* (11.54)	*68.25* (10.36)	−0.85 (0.39)
Social Problems	62.24 (7.72)	61.40 (6.43)	64.05 (9.89)	−1.28 (0.21)
Thought Problems	61.89 (8.80)	61.33 (8.91)	63.10 (8.65)	−0.74 (0.46)
Composite and Total Scales				
Internalizing Problems	*63.90* (9.11)	*62.72* (8.95)	**66.45** (9.12)	−1.53 (0.13)
Externalizing Problems	*60.63* (9.95)	*60.40* (10.73)	*61.15* (8.22)	−0.28 (0.78)
Total Problems	*63.87* (9.04)	*63.98* (9.22)	*63.65* (16.04)	0.10 (0.92)

Note. For syndrome scales: Scores in **bold** indicate the clinical range (T-score ≥ 70), and scores in *italics* indicate the clinical borderline range (T-score between 65 and 69). For composite and total scales: Scores in **bold** indicate the clinical range (T-score ≥ 64), and *italicized* scores indicated the clinical borderline range (T-score between 60 and 63).

**Table 2:** Means and Standard Deviations of T-scores for CBCL Syndrome Scales by Level of Sensory Processing Alteration, with Univariate F Value and Post Hoc Comparisons.

Level of Sensory Processing Alteration						
	A. No Alteration (n = 27)	B. Mild (n = 11)	C. Moderate (n = 6)	D. Severe (n = 19)		
CBCL	M (SD)	M (SD)	M (SD)	M (SD)	F(3, 59)	Post hoc/d
Anxious/Depressed	62.74 (9.61)	61.55 (10.95)	62.67 (7.31)	**70.05** (10.34)	2.66	
Withdrawn/Depressed	57.59 (8.20)	57.64 (6.25)	62.67 (13.62)	*67.37* (8.66)	5.46 **	A < D */−1.15
Somatic Complaints	58.15 (6.40)	60.82 (6.42)	59.33 (5.72)	63.53 (10.86)	1.73	
Rule-Breaking Behavior	56.37 (7.08)	57.73 (5.68)	60.17 (8.50)	60.79 (6.70)	1.71	
Aggressive Behavior	59.89 (8.75)	*65.18* (11.34)	64.83 (5.19)	*65.74* (8.08)	2.04	
Attention Problems	61.70 (9.18)	63.73 (8.49)	**74.00** (12.70)	**72.53** (11.25)	5.68 **	A < D */−1.05
Social Problems	59.67 (5.11)	59.91 (10.22)	63.83 (7.00)	*66.74* (7.78)	4.10	
Thought Problems	59.26 (7.40)	58.82 (9.08)	62.17 (7.78)	*67.32* (8.83)	4.23	

Note. A Bonferroni correction was applied to adjust for multiple comparisons across eight one-way ANOVAs, resulting in an adjusted significance threshold of α = 0.006 (0.05/8). Post hoc comparisons between the four groups were Bonferroni-adjusted for six pairwise comparisons, resulting in an adjusted significance threshold of α = 0.008 (0.05/6). * *p* < 0.008; ** *p* < 0.006. Scores in **bold** indicate values in the clinical range (T-score ≥ 70); *italicized* scores indicate clinical borderline values (T-score between 65 and 69).

**Table 3:** Means, Standard Deviation, and Univariate F Values for CBCL Internalizing, Externalizing, and Total Problems Scales by Level of Sensory Processing Alteration.

Level of Sensory Processing Alteration						
	A. No Alteration (n = 27)	B. Mild (n = 11)	C. Moderate (n = 6)	D. Severe (n = 19)		
CBCL	M (SD)	M (SD)	M (SD)	M (SD)	F(3, 59)	Post Hoc/d
Internalizing Problems	*60.56* (8.21)	*61.64* (9.21)	*63.83* (7.22)	**70.00** (8.23)	5.14 *	A < D **/−1.14
Externalizing Problems	56.59 (10.96)	*62.64* (9.22)	**64.17** (6.80)	**64.11** (7.96)	2.90	
Total Problems	*60.78* (8.84)	58.64 (20.94)	**68.33** (4.76)	**69.89** (5.59)	3.79 *	A < D +/−1.23

Note. A Bonferroni correction was applied to adjust for multiple comparisons across three one-way ANOVAs, resulting in an adjusted significance threshold of α = 0.017 (0.05/3). Post hoc comparisons between the four groups were Bonferroni-adjusted for six pairwise comparisons, resulting in an adjusted significance threshold of α = 0.008 (0.05/6). + *p* = 0.044; * *p* < 0.017; ** *p* < 0.008. Scores in **bold** indicate values in the clinical range (T-score ≥ 64); *italicized* scores indicate clinical borderline values (T-score between 60 and 63).

## Data Availability

The data supporting the reported results of this study are not publicly available due to privacy and ethical restrictions. Data can be made available upon reasonable request from the corresponding author, subject to the approval of the relevant ethical committee.
